# Managing high-risk behaviours and challenges to prevent housing loss in permanent supportive housing: a rapid review

**DOI:** 10.1186/s12954-023-00873-z

**Published:** 2023-09-29

**Authors:** Nick Kerman, Sean A. Kidd, Christina Mutschler, John Sylvestre, Benjamin F. Henwood, Abe Oudshoorn, Carrie Anne Marshall, Tim Aubry, Vicky Stergiopoulos

**Affiliations:** 1 Centre for Addiction and Mental Health, Toronto, ON Canada; 2 Department of Psychiatry, University of Toronto, Toronto, ON Canada; 3 School of Psychology, University of Ottawa, Ottawa, ON Canada; 4 Centre for Research on Educational and Community Services, University of Ottawa, Ottawa, ON Canada; 5 Suzanne Dworak-Peck School of Social Work, University of Southern California, Los Angeles, CA USA; 6 Arthur Labatt Family School of Nursing, Western University, London, ON Canada; 7 School of Occupational Therapy, Western University, London, ON Canada

**Keywords:** Permanent supportive housing, Housing first, Risk management, Mental illness, Safety, Violence, Overdose, Apartment takeovers, Fire, Review

## Abstract

Permanent supportive housing is an effective intervention for stably housing most people experiencing homelessness and mental illness who have complex support needs. However, high-risk behaviours and challenges are prevalent among this population and have the potential to seriously harm health and threaten housing tenures. Yet, the research on the relationship between high-risk issues and housing stability in permanent supportive housing has not been previously synthesized. This rapid review aimed to identify the housing-related outcomes of high-risk behaviours and challenges in permanent supportive housing settings, as well as the approaches used by agencies and residents to address them. A range of high-risk behaviours and challenges were examined, including risks to self (overdose, suicide/suicide attempts, non-suicidal self-injury, falls/fall-related injuries), and risks to multiple parties and/or building (fire-setting/arson, hoarding, apartment takeovers, physical/sexual violence, property damage, drug selling, sex trafficking). The search strategy included four components to identify relevant academic and grey literature: (1) searches of MEDLINE, APA PsycINFO, and CINAHL Plus; (2) hand searches of three journals with aims specific to housing and homelessness; (3) website browsing/searching of seven homelessness, supportive housing, and mental health agencies and networks; and (4) Advanced Google searches. A total of 32 articles were eligible and included in the review. Six studies examined the impacts of high-risk behaviours and challenges on housing tenancies, with overdose being identified as a notable cause of death. Twenty-six studies examined approaches and barriers to managing high-risk behaviours and challenges in PSH programs. These were categorized into eight types of approaches: (1) clinical, (2) relational/educational, (3) surveillant, (4) restrictive, (5) strategic, (6) design-based, (7) legal, and (8) self-defence. Consistent across all approaches was a lack of rigorous examination of their effectiveness. Further, some approaches that are legal, restrictive, surveillant, or strategic in nature may be used to promote safety, but may conflict with other program objectives, including housing stability, or resident empowerment and choice. Research priorities were identified to address the key evidence gaps and move toward best practices for preventing and managing high-risk behaviours and challenges in permanent supportive housing.

## Introduction

Permanent supportive housing (PSH) is a best practice intervention for stably housing people experiencing homelessness and mental illness who have complex support needs [1–3]. PSH involves the provision of permanent affordable housing, along with community-based mental health recovery-oriented supports, such as intensive case management or assertive community treatment. Research has demonstrated that 80–90% of people remain stably housed in PSH after up to six years [1, 4–9]. Yet, supporting people’s mental health recovery journeys can be challenging and there remains a small group of individuals who experience difficulties in PSH that can result in housing loss, relocation, recurrent homelessness, or rehospitalization [10–13]. Given the deleterious effects of housing loss among people with mental illness and histories of homelessness, it is critical to identify the risk factors that may lead to negative outcomes in PSH.

A sizeable body of research over the past decade has focused on predictors of housing outcomes in PSH. However, these studies have yielded limited evidence on the factors associated with PSH housing loss. In an early examination of data from a multisite randomized controlled trial of Housing First, an intervention often delivered as PSH, findings were only able to predict 3.8% of the variance in housing instability outcomes after 12 months using sociodemographic, clinical, and housing history variables [14]. Subsequent analyses from the same trial with a more stringent definition of housing stability and set of predictors produced an improved model, but ultimately yielded the same conclusion: Although certain individual characteristics are risks factors associated with difficulties establishing housing stability, the researchers concluded that it was not possible to accurately predict who would be unsuccessful in Housing First after 24 months [15]. Studies examining associations between service use and housing stability have also produced relatively small effect sizes [16]. An implicit assumption of these studies is that individual characteristics and behaviour patterns can be used to predict a trajectory of future housing stability problems. However, a person’s housing stability is dynamically shaped by their housing and supports, as well as the broader environment [17]. These contextual factors are only partially captured in research examining predictors of housing outcomes in PSH. In particular, sudden, unplanned, and acute events that may alter a housing trajectory have not been studied. Further, the interventions used by PSH service providers, either successfully or unsuccessfully, to mitigate the potential harms of such events have not been thoroughly examined in research on PSH housing outcomes. Accordingly, acute events and the accompanying risk management approaches used by PSH service providers may hold promise for potentially identifying at-risk individuals and intervening to prevent housing loss.

A range of high-risk behaviours and challenges may seriously harm the health of residents and threaten their housing tenures in PSH. These may include risks to self (e.g., overdose, suicide attempts, non-suicidal self-injury, and falls), or risks to multiple parties and buildings (e.g., fires, hoarding, apartment takeovers, violence, property damage, drug selling, sex trafficking). Some of these incidents may also involve PSH residents being victimized by other people. High-risk behaviours and challenges are prevalent among people with mental illness, substance use problems, and histories of homelessness, and in PSH settings. For example, in an examination of over 12,000 supportive housing applicants in Toronto, Canada, 20.3% had a history of suicide attempts, 17.7% were perpetrators or victims of physical assault, 14.7% had engaged in non-suicidal self-injury, 8.0% had fire safety concerns, 7.9% had damaged property, and 5.9% engaged in hoarding behaviours [18]. Another study with a more rigorous observational assessment found that 18.5% of formerly homeless individuals in PSH exhibited hoarding behaviours [19]. Further, in a study of supportive housing programs for formerly homeless older adults aged 45–80 years, most have experienced a fall in the past year, many of which resulted in a serious injury requiring medical care [20]. The overdose crisis has also disproportionally affected homeless and precariously housed populations. In San Francisco, overdoses were found to be nearly twenty times higher among residents of single room occupancy hotels, including some supportive housing programs, than non-single room occupancy residents [21]. Given both their prevalence and severity, many high-risk behaviours and challenges are burdensome for service providers in the supportive housing sector to manage and may be important intervention targets [22, 23]. These types of problems can also threaten program fidelity and sustainability of PSH programs due to loss of key relationship connections and knowledge among support teams [24, 25].

Several studies have examined the effects of PSH on high-risk behaviours and challenges. Housing First was found to reduce violent and nonviolent victimization, whereas the intervention had minimal effects on suicide attempt rates [26–30]. Yet, there are key evidence gaps with regard to other high-risk behaviours and challenges. For example, few studies have examined severe substance use-related harms, including overdoses, in PSH [31]. Other housing challenges, such as hoarding and apartment takeovers, have also not been studied in the context of PSH. Further, the extent to which high-risk issues affect housing stability in PSH has not been previously synthesized. As the research on high-risk behaviours and challenges suggests that some of these types of issues may be preventable or modifiable in the context of PSH using evidence-based approaches and best practices, a rapid review was undertaken to understand the practices that PSH programs use to manage high-risk behaviours and challenges, and the effectiveness of these approaches.

This rapid review aimed to identify the approaches and barriers to managing high-risk behaviours and challenges in supportive housing settings, with a focus on how these issues affect housing tenancies. Rapid reviews provide a streamlined approach to synthesizing evidence that can be efficiently disseminated to and used by sectoral decision-makers and service providers [32]. A rapid review was selected given the ongoing COVID-19 pandemic, worsening overdose crisis, affordable housing shortages, and inaccessible mental health services in many communities, which have exacerbated service delivery challenges in PSH settings [22, 33]. In this context, a timely synthesis of evidence can produce needed information on research gaps and inform service delivery approaches. The two research questions were: (1) What impacts do high-risk behaviours and challenges have on housing tenancies in PSH? and (2) What are the approaches and barriers to managing high-risk behaviours and challenges in PSH programs? For this rapid review, high-risk behaviours and challenges were defined as any critical events or serious behaviours that are potentially life-threatening and/or jeopardize a person’s housing tenure. The latter may be due to eviction or other causes, such as prolonged hospitalization, justice system involvement, or new support needs caused by an injury that cannot be met by individuals’ current supportive housing programs. The rapid review was not prospectively registered.

## Method

This rapid review followed guidelines by King and colleagues [34], with additional considerations for grey literature searching by Godin and colleagues [35]. Two sets of high-risk behaviours and challenges were examined: (1) risks to self (overdose, suicide/suicide attempts, non-suicidal self-injury, falls/fall-related injuries); and (2) risks to multiple parties and/or building (fire-setting/arson, hoarding, apartment takeovers, physical/sexual violence, property damage, drug selling, sex trafficking). Additional high-risk issues not identified at the outset of the review were also considered. A PICO framework was used to further establish review parameters, define key terms, and inform the search strategy (Table 1).

**Table 1:** PICO framework

*P*	Population	People exiting homelessness
		People with mental illness and/or who use substances
*I*	Intervention	PSH with onsite or offsite supports for single adults
		Alignment with a Housing First approach was not required
		Single room occupancy models were included if they offered some form of supportive services
*C*	Comparison	Not applicable
*O*	Outcome	Primary research question: approaches to managing high-risk behaviours and challenges used by service providers; service providers’ experiences in supporting residents with high-risk behaviours and challenges; residents’ experiences of high-risk behaviours and challenges in PSH
		Secondary research question: housing retention and loss; returns to homelessness; death

*PSH* Permanent supportive housing

Three academic databases were subsequently searched on November 14, 2022: (1) MEDLINE, (2) APA PsycINFO, and (3) CINAHL Plus. The following string of keywords was used: (homeless* OR mental illness OR mental disorder* OR psychiatric disorder OR substance use disorder OR drug* OR alcohol OR dual diagnosis OR dually diagnosed OR concurrent disorder*) AND (Housing First OR Pathways to Housing OR supportive housing OR supported housing) AND (suicid* OR self-harm OR self-injur* OR fire OR arson OR pyro* OR hoard* OR overdose OR poisoning OR toxicity OR adverse OR withdrawal OR intoxicat* OR drunk* OR inebriat* OR violen* OR assault* OR takeover* OR unwanted OR unwelcome OR cuckooing OR risk OR injur* OR death OR dying OR died OR fall* OR traffick* OR sexual exploitation OR property damage OR property offense* OR drug dealing OR drug trade OR drug selling). A multi-purpose field search was used with the MEDLINE and APA PsycINFO databases. Three journals with aims specific to housing and homelessness that are partially or not indexed in the three academic databases were also hand searched: (1) European Journal of Homelessness, (2) International Journal on Homelessness, and (3) Journal of Social Distress and Homelessness.

Grey literature was searched using a modified search strategy informed by Godin and colleagues [35]. This included: (1) website browsing/searching and (2) Advanced Google searches. Seven websites of homelessness, supportive housing, and mental health agencies and networks (Australian Alliance to End Homelessness, Canadian Alliance to End Homelessness, Corporation for Supportive Housing, FEANTSA, The Homeless Hub, National Alliance to End Homelessness, Substance Abuse and Mental Health Services Administration) were searched between November 2022-January 2023. An abbreviated list of keywords was used for the website and Advanced Google searches. Up to 200 consecutive records were reviewed in the Advanced Google searches.

Articles in both the academic database and grey literature searches were eligible for inclusion if they: (1) had findings specific to one or more high-risk behaviours and challenges in the context of PSH, which were linked to either personal experiences of residents, support approaches or experiences of service providers, or housing tenure (including death); (2) were a peer-reviewed journal article, book or book chapter, or technical report; (3) involved original research, case study, a review, or program evaluation; and (4) were published between January 1, 1992-October 31, 2022 (advanced publication articles were permitted). Exclusion criteria were: dissertations, conference abstracts, newspaper media, and blogs; studies examining transitional housing programs; and studies examining supportive housing for families or individuals experiencing interpersonal violence.

Academic articles were first screened by the lead author for relevance at the title and abstract levels. A highly conservative approach for exclusion was used during the screening phase so that all articles with slight applicability to the review were retained and further assessed. A full-text review was then completed to determine and summarize relevant findings from the articles that met the review eligibility criteria. A similar approach was used with the grey literature searches. Document titles and any accompanying summarizations were screened. A full-text review of potentially relevant documents was then completed. The lead author performed the searches, screening, and full-text reviews. A co-author (CM) reviewed 15% of the articles’ data extractions in the full-text reviews for accuracy, which demonstrated high consistency in assessments. Eligible articles were then narratively synthesized, and approaches for addressing high-risk behaviours and challenges were categorized thematically. The lead author completed the initial labelling and defining of the thematic categories, which were then reviewed by and discussed with a co-author (CM), producing consensus assessments.

## Results

### Description of articles in rapid review

**Fig. 1:**
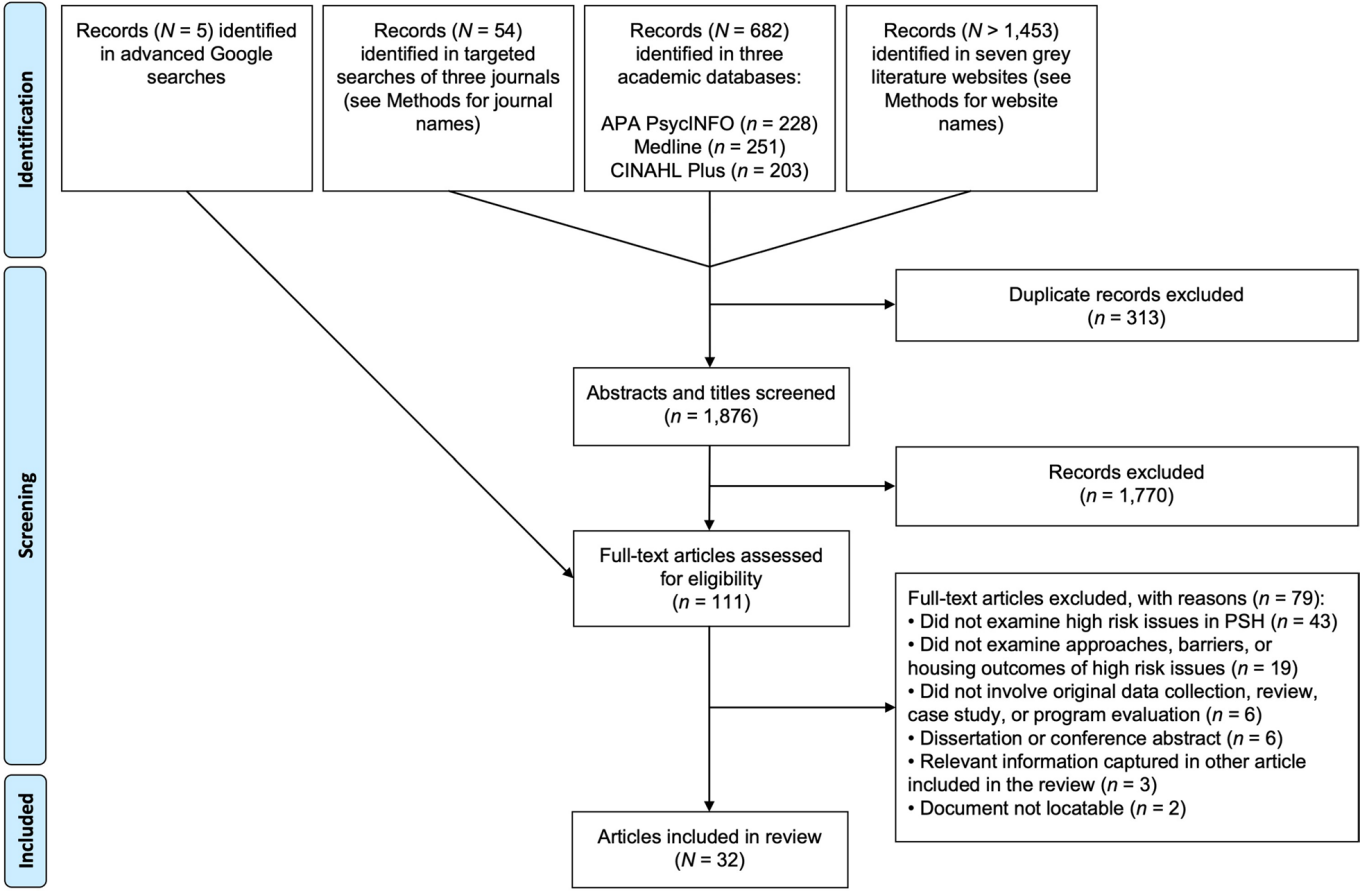
Search summary and article selection process. *Note*. Records could not be enumerated for a part of the grey literature website search that involved browsing relevant webpages. Hence, *N* > 1,453

A total of 32 articles were eligible and included in the review (Fig. 1). Six studies examined the impacts of high-risk behaviours and challenges on housing tenancies (research question (1), whereas twenty-six studies examined approaches and barriers to managing high-risk behaviours and challenges in PSH programs (research question 2). Most studies were conducted in North America, with 15 from the United States and 12 from Canada. Two articles were from a single study in France, two articles were from a single study in Australia, and one article was from Norway. There was variability in PSH models across the studies and details about support models were inconsistent, making it unfeasible to examine differences in findings by program model and philosophy (Table 2).

**Table 2:** Summary of articles in rapid review

Year	References	Article type	Location	Housing and support model	High-risk issues examined
2022	Ivsins et al. [36]	Qualitative study	Vancouver, Canada	PSH program for people with physical, mental health, and substance use problems; primary care and substance use services (opioid agonist therapy and prescribed safer support) available onsite, as well as a drug consumption site and managed alcohol program	Overdose
2022	Nixon & Burns [37]	Qualitative study	Western Canada	70-bed PSH program for older adults (> 55 years) with experiences of homelessness and complex health problems; health services provided onsite, including managed alcohol and tobacco programs, and onsite opioid agonist therapy dispensing	Falls caused by alcohol intoxication
2022	Wood et al. [38]	Program evaluation	Perth, Australia	Housing First program with a collaborative support model that involved over 30 participating organizations and had fairly good adherence to Australian Housing First principles	Property damage; interpersonal threats
2021	Bardwell et al. [39]	Qualitative study	Vancouver, Canada	Women-only, single room occupancy-based supportive housing program with wireless overdose response button system installed in residential units	Overdose; violence
2021	Chavez et al. [40]	Study protocol	Large Midwestern city, United States	Housing First for youth (model not described) with adjunct intervention of cognitive therapy for suicide prevention	Suicide attempts
2021	Corporation for Supportive Housing [41]	Cross-sectional survey	New York State, United States	Various supportive housing programs, including scattered- and single-site models	Overdose
2021	Milburn et al. [42]	Exploratory mixed-methods study	Los Angeles, United States	Various PSH programs, including scattered- and single-site models	Trespassing; uninvited guests; weapons possession
2021	Roebuck et al. [43]	Implementation and outcome program evaluation	Ottawa, Canada	Scattered-site, condominium-based Housing First program, with slight deviations from the Pathways Housing First model	Apartment takeovers
2021	Tinland et al. [44]	Randomized controlled trial	Paris, Marseille, Toulouse, and Lille, France	Scattered-site Housing First with assertive community treatment for people with Schizophrenia or Bipolar Disorder and fidelity to the Pathways Housing First model	Suicide and overdose as causes of death
2020	Vallesi et al. [45]	Program evaluation	Perth, Australia	Housing First program aligned with the core principles of European Housing First	Threatening neighbours and violent relationships; apartment takeovers
2019	Bardwell et al. [46]	Qualitative study	Vancouver, Canada	Single room occupancy hotels with adjunct peer-led overdose response intervention	Overdose
2019	Brar et al. [47]	Case report	Vancouver, Canada	Single room occupancy-based supportive housing program	Overdose
2019	Friesinger et al. [48]	Ethnographic qualitative study	Southern Norway	Seven supported housing programs for people with mental illness of variable size; mix of single-site buildings and small houses	Fire
2019	Katz et al. [49]	Randomized controlled trial	Moncton, Montreal, Toronto, Winnipeg, Vancouver, Canada	Housing First with fidelity to the Pathways Housing First model	Suicide attempts
2018	Addictions & Mental Health Ontario et al. [50]	Multiple case study	Ontario, Canada	Various supportive housing programs, including two single-site models that employ a hoarding specialist on the support team	Hoarding
2018	Gutman et al. [51]	Exploratory mixed-methods study	New York City, United States	Two supportive housing programs for formerly homeless adults	Falls
2018	Henwood et al. [52]	Ethnographic qualitative study	Los Angeles, United States	Various PSH programs, including scattered- and single-site models	Guest-based victimization, including violence and stalking; drug selling and availability
2018	Rhenter et al. [53]	Qualitative study	Paris, Marseille, Toulouse, and Lille, France	Scattered-site Housing First with assertive community treatment for people with Schizophrenia or Bipolar Disorder and fidelity to the Pathways Housing First model	Violence
2018	Tiderington [54]	Multi-method qualitative study	Large urban city, United States	Two scattered-site PSH programs, one of which had a transitional housing feeder program	Apartment takeovers
2017	Chang [55]	Docent method qualitative study	San Francisco, United States	Ten supportive housing buildings operated by an agency that uses a Housing First approach	Drug selling and availability
2017	Cusack & Montgomery [56]	Retrospective administrative data analysis	United States	HUD-VASH PSH program for veterans	Suicide and self-injury
2017	The Dream Team [57]	Community-based participatory, multi-methods research study	Toronto, Canada	Non-specific (participants lived in carious supportive housing programs or social housing)	Apartment takeovers
2016	Kriegel et al. [58]	Explanatory sequential mixed-methods study	California, United States	Forensic and non-forensic full-service partnerships, with variable fidelity to Pathways Housing First	Neighbourhood crime and drug availability; threatening neighbours
2015	Henwood, et al. [59]	Observational study	Philadelphia, United States	Pathways Housing First program	Substance and fire-related causes of death
2014	Distasio et al. [60]	Multiple case study	Canada	Various supportive housing programs	Violence; pedophilia; property damage; hoarding; unwanted visitors
2014	Silva et al. [61]	Description of adverse events and responses in randomized controlled trial	Moncton, Montreal, Toronto, Winnipeg, Vancouver, Canada	Housing First with fidelity to the Pathways Housing First model	Stovetop fires; violence; weapon offences; uninvited guests
2013	Collins et al. [62]	Nonrandomized controlled trial	Seattle, United States	Single-sited Housing First program for chronically homeless adults with severe alcohol use problems	Hostility
2012	Krüsi et al. [63]	Qualitative study	Unspecified city in British Columbia, Canada	Two minimal-barrier, high-tolerance supportive housing programs for chronically homeless women engaged in sex work	Violence; rape
2009	Lee et al. [64]	Cross-sectional study	Philadelphia, United States	Supportive independent living program for people with serious mental illness that required sobriety and treatment compliance	Neighbourhood crime against people and property
2009	Pearson et al. [65]	Exploratory, longitudinal study	New York City, Seattle, San Diego, United States	Three Housing First programs serving people experiencing homelessness and mental illness that are operated by three different agencies (Pathways Housing First, DESC, and REACH)	Interpersonal abuse; property damage
2005	Campanelli et al. [66]	Program description and evaluation	New York City and Montgomery County, United States	Housing with various structures and supports for people with mental illness	Violence; arson
1996	Sohng [67]	Process evaluation	Urban county in Washington State, United States	Two-bedroom apartment-based housing program with onsite supports for older adults with mental illness	Sexual and verbal aggression

PSH: permanent supportive housing

A range of high-risk behaviours and challenges were examined across the two research questions. These included: apartment takeovers, trespassing, and uninvited guests (*n* = 7); overdose and substance-caused fatalities (*n* = 7); non-specified violence and hostility (*n* = 7); suicide and self-injury (*n* = 5); fires and arson (*n* = 4); interpersonal threats and abuse, including from neighbours (*n* = 4); drug availability and selling (*n* = 3); property damage (*n* = 3); sexual violence, including assault, harassment, and stalking (*n* = 3); falls and fall-related injuries (*n* = 2); hoarding (*n* = 2); neighbourhood crime toward people and property (*n* = 2); and weapons possession (*n* = 2). Pedophilia and verbal aggression were each examined in a single article.

### Outcomes of high-risk behaviours and challenges on housing tenure

Six studies examined housing-related outcomes associated with various high-risk behaviours and challenges in PSH (Table 3). Four studies examined correlates of PSH exits. Greater hostility, as measured by distress caused by emotion dysregulation, interpersonal arguments, and violent urges, was significantly associated with an increased likelihood of leaving a single-site Housing First program for chronically homeless adults with severe alcohol use problems [62]. In contrast, suicide or self-injury, neighbourhood crime (offences against property and people), interpersonal abusiveness, and property damage were not significantly associated with PSH exits in three other studies [56, 64, 65]. Two studies examined causes of death in Housing First. In a randomized controlled trial of Housing First conducted in France, overdose was the leading cause of death (*n* = 8, 34.8%) among the 23 residents of the intervention group who passed away – a rate higher than the treatment as usual group, which had no overdose deaths [44]. In an earlier observational study of 41 residents who died while participating in a Housing First program, a smaller proportion of deaths were the result of alcoholism or drug intoxication (*n* = 4, 9.8%) in comparison to the French study, and no residents died from fire-related causes [59].

**Table 3:** Housing-related outcomes associated with high-risk behaviours and challenges in permanent supportive housing

Year	Authors	High-risk issues examined	Housing outcomes examined	Findings
2021	Tinland et al. [44]	Suicide and overdose as causes of death	Death	34.8% (*n* = 8) of Housing First resident deaths were from overdose, whereas no participants in treatment as usual group died of overdose 13.1% (*n* = 3) of Housing First resident deaths were from suicide, whereas 9.1% (*n* = 1) in treatment as usual group died of suicide
2017	Cusack & Montgomery [56]	Suicide and self-injury	Exits due to incarceration and returns to homelessness	Suicide or self-injury was not significantly associated with either supportive housing exits due to incarceration or returns to homelessness
2015	Henwood et al. [59]	Substance and fire-related causes of death	Death	9.8% (*n* = 4) of Housing First resident deaths were from alcoholism or drug intoxication No Housing First residents died from fire-related causes
2013	Collins et al. [62]	Hostility	Housing retention	Greater hostility was significantly associated with increased likelihood of leaving the Housing First program
2009	Lee et al. [64]	Neighbourhood crime against people and property	Supportive housing departures	Neighbourhood crime level was not significantly associated with departures from supportive housing
2009	Pearson et al. [65]	Interpersonal abuse; property damage	Housing tenure	No significant differences were found between leavers and stayers in interpersonal abusiveness and property damage

### Approaches to managing high-risk behaviours and challenges

The approaches to managing high-risk behaviours and challenges in PSH programs, as described in 26 studies, are summarized in Table 4. Each approach was also categorized as being clinical, relational/educational, surveillant, restrictive, strategic, design-based, legal, or self-defence (see Table 5 for descriptions and examples of each type). Almost all studies (*n* = 25) examined organizational and support approaches to managing high-risk behaviours and challenges, with five studies also describing how PSH residents responded to such problems. These are described in more detail below.

**Table 4:** Approaches to managing high-risk behaviours and challenges in permanent supportive housing

Year	Authors	High-risk issues examined	Risk management approach (type category)	Approach effectiveness and perceptions
2022	Ivsins et al. [36]	Overdose	Development and access to an onsite supervised consumption room and safer supply program (clinical and design-based)	Qualitative experiences of residents, including non-use of the consumption room due to interpersonal safety concerns and preference to use alone, and feelings of safety and less use of street-acquired drugs due to access to safer supply
2022	Nixon & Burns [37]	Falls caused by alcohol intoxication	Increased monitoring of residents (surveillant) Removal of excess alcohol (restrictive)	Not meaningfully examined
2022	Wood et al. [38]	Property damage; interpersonal threats	Liaise and advocate with housing providers about damage debts (relational/educational) Develop safety strategies (clinical) Provide support to apply for restraining orders (legal)	Not examined
2021	Bardwell et al. [39]	Overdose; violence	Installation of overdose response button technology in residential units (design-based)	Qualitative experiences of use by residents, including minimal use of the technology as intended, but use for other emergencies, such as gender-based violence
2021	Chavez et al. [40]	Suicide attempts	10-session cognitive therapy for suicide prevention (clinical)	Outcome analyses are planned, but not yet examined
2021	Corporation for Supportive Housing [41]	Overdose	Naloxone training for staff (clinical)	Not examined
2021	Milburn et al. [42]	Trespassing; uninvited guests; weapons possession	Use of a security team to patrol buildings (surveillant) Possession of weapons for self-protection (self-defence)	Qualitative experiences of residents, including perceptions that patrols were infrequent and inconsistent, yielding minimal effects on trespassing
2021	Roebuck et al. [43]	Apartment takeovers	Acquisition of housing units not located on the ground floor (strategic) Case managers support residents to develop healthy support networks and set boundaries with other people (relational/educational)	Not meaningfully examined
2020	Vallesi et al. [45]	Threatening neighbours and violent relationships; apartment takeovers	Transfer residents to more suitable accommodations (strategic) Staff intervene with uninvited guests on behalf of residents (relational/educational)	Not meaningfully examined
2019	Bardwell et al. [46]	Overdose	Development of a tenant-led overdose response team to provide naloxone training and distribution (clinical)	Qualitative experiences of residents and staff, demonstrating intervention acceptability and empowerment; however, residents also reported increased emotional distress due to the severity of the overdose crisis and need to administer naloxone to friends
2019	Brar et al. [47]	Overdose	Implementation of onsite injectable opioid agonist therapy (clinical)	Drug use outcomes over nine months, but no findings specific to overdose
2019	Friesinger et al. [48]	Fire	Use of fire detection, alarm, and extinguisher systems (design-based) Staff check-ins (surveillant) Prohibition of lighters for residents with histories of causing fires (restrictive) Automatic timed switches to turn off stoves and temperature sensors (design-based)	Qualitative experiences of staff and residents, including disablement or misuse of fire safety measures (e.g., bringing in unapproved visitors via emergency and fire exit doors), and concerns about surveillance and restrictiveness
2019	Katz et al. [49]	Suicide attempts	Use of the MINI Suicidality Subscale as a tool to predict suicide attempts (clinical)	Instrument had high predictive validity of suicide attempts among people experiencing homelessness and mental illness over a 24-month period
2018	Addictions & Mental Health Ontario et al. [50]	Hoarding	Use of a hoarding specialist on support teams (clinical)	Not examined
2018	Gutman et al. [51]	Falls	Standardized safety assessment conducted during home visits (clinical)	Identification of environmental fall risks
2018	Henwood et al. [52]	Guest-based victimization, including violence and stalking; drug selling and availability	Residents’ isolation at home to avoid drug use (restrictive) Enforcement of visitation program rules (restrictive)	Qualitative experiences of residents including risk of loneliness associated with the isolation approach and preference among women to be alone due to past traumas involving violence and stalking
2018	Rhenter et al. [53]	Violence	Residents’ engagement in sedentary behaviours to protect against street violence (restrictive)	Not examined
2018	Tiderington [54]	Apartment takeovers	Single-use language in occupancy policies (restrictive) Discouragement of social relationships (restrictive) Surveillance of apartment units and use of drop-ins (surveillant)	Not examined
2017	Chang [55]	Drug selling and availability	Widespread presence of security cameras in buildings and surrounding neighbourhood (surveillant)	Not meaningfully examined
2017	The Dream Team [57]	Apartment takeovers	Police, security, support worker, or family/friend involvement (relational/educational and legal) Use of screening tools to assess risk for apartment takeovers (clinical)	Qualitative experiences of residents, which indicated that police involvement was a last resort due to mistrust, concerns about effectiveness, and fears about housing loss
2016	Kriegel et al. [58]	Neighbourhood crime and drug availability; threatening neighbours	Courts exert influence on release decisions based on housing models and location (legal, restrictive, and strategic) Selectiveness by service providers as to where to appropriately house residents (strategic)	Implementation challenges described, such as tension for service providers between court requirements and Housing First principles
2014	Distasio et al. [60]	Violence; pedophilia; property damage; hoarding; unwanted visitors	Exclusion policies for applicants with histories of violence and pedophilia (restrictive) Behaviour agreements that outline rules, rights, and consequences (relational/educational) Increased visitation to residents’ homes (surveillant) Eviction notices as a “wakeup call” (strategic) Retention of leaseholder rights by PSH agency, with units sublet to residents, to enable staff to enter units and expel guests (strategic) Peer support focusing on the challenges of visitor management (relational/educational) Transitioning residents to a shelter for respite (strategic)	Not meaningfully examined
2014	Silva et al. [61]	Stovetop fires; violence; weapon offences; uninvited guests	Installation of motion detectors on stoves (design-based) Police involvement and pressing charges (legal) Provision of education and mentorship to residents about who should be allowed to enter apartments (relational/educational)	Motion detection technology on stoves decreased stovetop fires; outcomes not examined for other two approaches
2012	Krüsi et al. [63]	Violence; rape	Women-only buildings (design-based) One-guest maximum policy and registration logs (restrictive and surveillant) Bad-date reports (strategic) Camera surveillance (surveillant) Staff and police involvement (relational/educational and legal)	Qualitative experiences of residents, with approaches generally being perceived positively and contributing to a sense of safety
2005	Campanelli et al. [66]	Violence; arson	Screening assessment for applicants with histories of violence and arson (clinical)	Tenancy outcomes of applicants accepted following the screening assessment were presented, but few details on why residents were no longer housed in the program
1996	Sohng [67]	Sexual and verbal aggression	Rehospitalization (clinical) Transition to respite care (clinical)	Not meaningfully examined

**Table 5:** Types of approaches to managing high-risk behaviours and challenges in permanent supportive housing

Type	Description	Examples
Clinical	Use of existing or augmentations to professional support services for the purpose of assessment and intervention	Establishment of specialized services, such as hoarding specialists and harm reduction supports Development and implementation of risk-related screening tools
Relational/educational	Use of working relationships and informational strategies between PSH staff, often case managers and other direct service providers, landlords, and residents to address high-risk behaviours and challenges	Advocacy with landlords about damage debts Provision of education and mentorship to residents about who should be allowed to enter apartments
Surveillant	Implementation of measures to monitor PSH residents and visitors	Installation of video cameras in and around PSH buildings Staff drop-ins on residents
Restrictive	Use of PSH policies and practices that limit program access, and the behaviours of residents and visitors, as well as choices made by residents to refrain from specific behaviours and locations	Program policies that exclude applicants with histories of high-risk issues Resident-initiated isolation in housing unit to avoid conflict, access to substances, or another type of threat
Strategic	Use of pragmatic strategies to reduce the likelihood of high-risk behaviours and challenges or facilitate their cessation	Placement of residents in non-first floor housing units to prevent apartment takeovers Transfer of residents in unsafe buildings to new housing
Design-based	Built environment and program design decisions and adaptations to reduce the risk of critical events	Installation of stovetop motion sensors to reduce fire risk Development of women-only PSH programs
Legal	Engagement with legal systems in response to high-risk behaviours and challenges	Pursuit of charges and justice system-based protections following offences Provision of emotional and practical support to report crimes
Self-defence	Actions initiated by residents for the purpose of self-protection	Acquiring and carrying weapons in response to safety concerns

*PSH* Permanent supportive housing

### Resident experiences with high-risk behaviours and challenges

Of the five studies examining how PSH residents responded to high-risk behaviours and challenges, two discussed avoidance of potential threats, including people and drugs [52, 53]. Gender-based violence was the focus of another study, which showed that women who experienced chronic homelessness and engaged in sex work accessed support from program staff and police, a combined relational/educational and legal approach, to address safety concerns [63]. A similar method was also described with apartment takeovers, with residents involving police, security, support workers, or family and friends to resolve the situation [57]. Although the study did not examine the effectiveness of this approach, police involvement was perceived to be a last resort due to mistrust, concerns about effectiveness, and fears about housing loss [57]. Lack of responsiveness by PSH programs to safety threats could also lead residents to consider self-defence strategies. A study of Black PSH residents in Los Angeles found that some carried weapons for self-protection, making this both a type of high-risk behaviour and a response to victimization [42].

### Organizational and support approaches

*Violence, aggression, and interpersonal threats and abuse* were a set of related high-risk behaviours and challenges for which a variety of preventive and reactive approaches were described. Restrictive approaches were commonly used to prevent violence, such as exclusion policies for PSH program applicants with histories of violence and enforcement of visitation program rules to prevent exploitation of residents by guests [52, 60]. There were mixed perceptions among residents of the latter strategy, though women with histories of abuse and sexual victimization viewed that the visitation rules created a sense of safety in their housing [52]. Strategic approaches typically focused on housing location, with service providers being selective about where to appropriately house residents or supporting their transfer to more suitable accommodations [38, 58]. Neither study examined the effectiveness of the strategic approaches. One other study examined a design-based intervention, overdose response buttons in residential units, with findings showing that this technology was used more often for other emergencies, such as violence, than the intended purpose [39]. Approaches could also be combined as part of a multifaceted safety model. For example, to prevent sexual violence in two supportive housing programs for chronically homeless women engaged in sex work, the organizations made use of women-only buildings (design-based); a maximum one-guest policy, registration logs, and security cameras (restrictive and surveillant); and bad-date reports (strategic) [63].

Legal and clinical approaches were also discussed in response to past or ongoing violence. Legal approaches involved case managers supporting residents to obtain restraining orders against threatening individuals, or programs pressing charges or pursuing eviction proceedings in response to violence [38, 60, 61]. The latter approaches highlight how attempts to manage risk may also counter efforts to sustain tenancies. Clinical approaches to addressing violence and aggression included: the use of screening assessments with prospective PSH applicants, the development of safety plans, and transfers of residents to other service settings (e.g., hospital, respite care) [38, 66, 67]. None of these studies measured the effectiveness of the legal or clinical approaches.

*Apartment takeovers and trespassing* were primarily addressed using relational/educational, strategic, and surveillant approaches. Relational/educational approaches involved PSH staff intervening directly (i.e., engaging and confronting uninvited guests) or indirectly (e.g., supporting residents to strengthen boundary-setting skills, offering peer support focused on visitor management) to problem-solve the issue [43, 45, 60]. Strategic approaches involved not acquiring ground floor housing units where there would be fewer barriers to apartment takeovers, retaining leaseholder rights by the PSH agency to permit direct intervention, and temporarily transitioning residents to shelters for respite as the problem is addressed [43, 60]. Surveillant approaches were used by security, who conducted patrols of PSH buildings to prevent trespassing, as well as by staff who tracked residents’ occupancy violations and dropped in on apartments unexpectedly to enforce visitor policies [42, 54]. Restrictive strategies to prevent apartment takeovers, such as single-use language in occupancy policies and discouragement of social relationships, were also identified in one study [54]. Use of screening tools to assess risk for apartment takeovers was a clinical approach that was proposed in one study but not studied [57]. Only one of the six studies examined outcomes associated with these approaches, with PSH residents perceiving that security patrols were ineffective in deterring trespassers due to inconsistency issues [42].

Five studies examined approaches to preventing and intervening with *overdoses*. Most of these involved clinical interventions, such as onsite supervised consumption rooms, opioid agonist therapy and safer supply programs, and naloxone training and distribution [36, 41, 46, 47]. Qualitative experiences associated with the approaches were examined in two studies; both of which were generally positive, but limitations with the interventions were also noted [36, 46]. The other two studies did not report outcomes specific to overdose [41, 47]. The fifth study found that overdose response buttons in residential units were used minimally by residents to report imminent drug use [39].

Approaches to preventing *fire and arson*, as described in three studies, varied. These included design-based strategies, such as fire detection alarms and technology to automatically turn off stoves [48, 61]. The latter approach was reported to reduce stovetop fires in one article, whereas some residents experienced this as disempowering in the other study. Surveillant and restrictive approaches were also used to promote fire safety, which residents experienced as privacy intrusions and potential triggers for paranoia [48]. One other study described a screening tool that assessed PSH applicants for past incidents of arson; the effectiveness of this clinical approach in supporting individuals with fire-setting histories and preventing reoccurrence was not discussed [66].

*Suicide* risk was the focus of two studies. One found that the MINI Suicidality Subscale was a valid tool for predicting suicide attempts among people experiencing homelessness and mental illness in a Housing First trial [49], whereas the other was a study protocol for a co-intervention involving cognitive therapy for suicide prevention in a Housing First for youth program [40].

Prevention of *falls* involved clinical (home visit safety assessment), surveillant (increased monitoring of intoxicated residents), and restrictive approaches (removal of alcohol during intoxication) [37, 51]. The structured safety assessment effectively identified environmental fall risks, but no other outcomes were reported for the fall prevention strategies.

Two studies described approaches for managing *drug selling and availability* risks in PSH buildings and the surrounding neighbourhood. One qualitative study described the widespread presence of security cameras, a surveillant approach [55], whereas another mixed-methods study highlighted how forensic Housing First programs had to navigate competing priorities of the court system that exerted its influence on release decisions based on residents’ substance use histories and the drug presence in communities (legal) [58].

Several approaches to addressing *property damage* were discussed in two studies, without describing outcomes. These included relational/educational interventions, such as advocacy with landlords related to damage debts, and surveillant approaches (increased visitation to residents’ homes to monitor for damages) [38, 60]. The use of a hoarding specialist was a clinical approach to managing *hoarding* described in one article [50] and program exclusion policies for PSH applicants with histories of *pedophilia* was identified in another [60]. Neither study discussed any relevant outcomes.

## Discussion

The rapid review findings demonstrate that a range of approaches are used to prevent and manage high-risk behaviours and challenges in PSH settings. The approaches were categorized into eight types, which were used in different ways or in combination to address various high-risk behaviours and challenges. Overdose was somewhat of an exception, as it was primarily addressed using clinical interventions. Consistent across all approaches was a lack of rigorous examination of their effectiveness. In studies that presented outcomes, these primarily focused qualitatively on the experiences of PSH staff and residents. Although the qualitative findings highlight key barriers with some approaches, the paucity of outcomes research represents a critical evidence gap that prevents the identification of evidence-based practices for addressing high-risk behaviours and challenges in PSH programs.

It is important to note that, beyond the PSH literature, there are only a few effective interventions for some high-risk behaviours and challenges, such as arson and fire-setting [68], and apartment takeovers [69]. PSH programs could be well-positioned for pilot interventions related to these high-risk issues, given the vulnerability of residents. In contrast, best practice interventions have been established for other high-risk behaviours and challenges, such as hoarding [70], suicidality [71], and overdose [72]. Although lessons can be drawn from this evidence base, the transferability of the approaches and potential implementation barriers warrant some cautiousness. For example, despite an onsite supervised consumption room being established in a PSH building, most residents continued to use drugs alone in their rooms [36]. Other studies highlighted how safety features in PSH units were misused, disabled, or used for alternative purposes [39, 48]. Thus, there is a need to not only identify effective practices and policies for preventing and managing high-risk behaviours and challenges in PSH but also to determine how acceptable these are to residents. Co-designing interventions with PSH residents may be beneficial for maximizing their utility and value.

Effective risk management approaches are a necessity for ensuring safety in PSH. Yet, these approaches require balance with other objectives of PSH programs so as to not become sites of social control [73]. An emphasis on safety and security may also conflict with other priorities. For example, legal approaches that involve initiation of eviction proceedings and police involvement could be used in response to violence and weapon offences [61]. Although PSH programs may take such actions as a final measure, these can threaten the resident’s housing stability. Accordingly, it is important that evictions from PSH use procedurally just processes and that residents be supported to obtain new housing in the absence of prolonged hospitalization or incarceration. These practices are necessary for balancing safety in PSH with a right to housing. Restrictive and surveillant approaches also have the potential to infringe on tenets of some PSH programs, such as individual choice, empowerment, and self-determination, and undermine privacy and mental health recovery [74]. Strategic approaches that involve relocating residents to new buildings in response to high-risk issues may similarly limit agency when such actions are misaligned with the preferences of residents. It is also important to note that PSH residents do not experience these strategies uniformly. For example, women with histories of trauma, abuse, and sex work appreciated the protection yielded from surveillant and restrictive approaches, leading to generally positive perceptions of these practices [52, 63]. This likely reflects the diversity of PSH residents and differences in their support needs based on past experiences, including trauma. Thus, the rapid review findings underscore the importance of engaging PSH residents in the development of risk management approaches, so that they may promote safety without impeding other program objectives and values.

Despite the prevalence of high-risk behaviours and challenges in PSH and their potential for serious consequences, only six studies have examined housing-related outcomes. Substance use-linked causes of death, including overdose, were reported as occurring in two studies of Housing First programs. These findings align with recent research that has identified overdose as a serious concern in supportive housing programs [23, 41]. More broadly, substance use problem severity has been identified as a risk factor associated with housing instability in PSH and continued connections to people who use substances may present eviction and apartment takeover risks [15, 75]. The latter findings highlight the complexity of social networks among people who use substances in PSH, as both potential sources of important support and risk [76, 77]. Greater integration of harm reduction services and peer support, as well as more landlord collaboration and education, may be beneficial for reducing preventing eviction risks and substance use-related harms, including overdose, in PSH [78–80]. Beyond substance use, studies in this review mostly produced non-significant results on the associations between high-risk behaviours and challenges, and exits from PSH. Conclusions are premature given the variation in studied issues and the preliminary state of the evidence, though the findings raise the prospect that high-risk behaviours and challenges can be effectively managed in PSH to prevent housing loss. Understanding how this can be done and documenting practice-based knowledge remains a critical need.

Given that there are key evidence gaps with regard to the prevention and management of high-risk behaviours and challenges in PSH, it is necessary to identify research priorities that have key implications for future practice and policy. First, few studies examined the outcomes of approaches to preventing and managing high-risk behaviours and challenges in PSH and, of the ones that did, most focused on the qualitative perceptions of program staff and residents. Thus, there is a need to investigate effective approaches for preventing and managing high-risk behaviours and challenges in PSH and the acceptability of these practices to residents. Second, six studies examined the housing outcomes associated with high-risk behaviours and challenges; however, analyses have mostly been descriptive or limited in scope. This raises the importance of examining if and how high-risk behaviours and challenges mediate the relationship between clinical characteristics and PSH housing outcomes. Third, research on staff training in risk management was notably absent from the review, with the exception of two studies that discussed naloxone training for overdose prevention. Future research is needed to identify the foundational training competencies for risk management in PSH settings. Fourth, screening and assessment tools were described or used for specific types of high-risk behaviours and challenges in four studies. Despite the dearth of research on risk assessment instruments, clinical assessment is a core component of PSH service delivery, which may include an examination of risk-related behaviours [81, 82]. More investigation is warranted into the risk assessment tools currently being used to assess high-risk behaviours and challenges in PSH, and the comprehensiveness and effectiveness of these instruments. Fifth, hoarding behaviours are prevalent among people with histories of homelessness, but approaches to addressing hoarding in PSH have been minimally examined, with no interventional studies having been conducted. A key research priority is to determine if evidence-based treatments for hoarding, such as Cognitive Behavioural Therapy, are effective for PSH residents and how feasible it is to deliver these interventions in these settings. Lastly, PSH models and philosophies may shape the types of risk management approaches used by programs. For example, PSH agencies that function as both the landlord and support team, as well as single-site programs, may experience greater tension in balancing the needs of the individual, other residents, and the building, leading to greater risk aversion on the part of the organization. It was not feasible to analyze how program models shaped the types of risk management approaches given the variability in PSH programs and populations presented in the rapid review articles. Because of this, future research is needed to determine how PSH models and philosophies affect the types of approaches used to prevent and manage high-risk behaviours and challenges.

There were several limitations to this rapid review. First, high-risk behaviours and challenges were defined as critical events or serious behaviours that had the potential for deleterious health and housing consequences. This high threshold may have omitted other key issues that threaten housing tenancies or are precursors to potential high-risk behaviours. Second, studies examining adjunct interventions in PSH that have implications for preventing high-risk behaviours and challenges, but which did not measure the specific outcomes of interest to the review, were excluded [83–85]. Nevertheless, these articles may describe additional approaches that could be beneficial for preventing high-risk issues and associated harms. Third, in clustering a range of high-risk behaviours and challenges into a single group, there is an underlying assumption that each of these behaviours and challenges have the potential to cause serious injury, death, or eviction. However, it is likely that some of these behaviours and challenges pose a higher risk of negative outcomes than others. Fourth, website browsing/searching for grey literature was restricted to well-known organizations and networks that offer resources to the supportive housing sector. As a result, relevant documents, especially technical reports of small program evaluations, not listed in these large website registries, may have been missed. Fifth, rapid reviews are not required to include a risk-of-bias assessment [34] and this review did not have one. Thus, some studies included in the review may have produced more methodologically rigorous findings than others. Nevertheless, very little evidence exists on the effects of high-risk behaviours and challenges on housing-related outcomes in PSH, regardless of study quality, making this is a critical area for future research.

## Conclusions

High-risk behaviours and challenges are prevalent among people with mental illness and histories of homelessness. This rapid review examined the housing-related outcomes of high-risk behaviours and challenges in PSH, and how agencies and residents addressed them. Findings showed that few studies have explored the relationship between high-risk behaviours and challenges, and housing outcomes in PSH, though overdose has been identified as a notable cause of death. As for how PSH programs manage risk, a range of approaches are used, yet their outcomes have also been minimally examined. The lack of evidence on outcomes prevents the identification of evidence-based practices for preventing and managing high-risk behaviours and challenges in PSH. Further, some approaches that are legal, restrictive, surveillant, or strategic in nature may be used to promote safety, but conflict with other PSH objectives, including housing stability, or resident empowerment and choice. Accordingly, there is a need to better understand if and how these approaches can be used in a person-centred and mental health recovery-oriented manner. Six research priorities were identified to address the key evidence gaps and move toward best practices for preventing and managing high-risk behaviours and challenges in PSH.

## Data Availability

Not applicable.

## Abbreviation

PSH: Permanent supportive housing
